# Spatio-Temporal Distribution of Injured Elephants in Masai Mara and the Putative Negative and Positive Roles of the Local Community

**DOI:** 10.1371/journal.pone.0071179

**Published:** 2013-07-30

**Authors:** Domnic Mijele, Vincent Obanda, Patrick Omondi, Ramón C. Soriguer, Francis Gakuya, Moses Otiende, Peter Hongo, Samer Alasaad

**Affiliations:** 1 Kenya Wildlife Service, Nairobi, Kenya; 2 Estación Biológica de Doñana, Consejo Superior de Investigaciones Científicas (CSIC), Sevilla, Spain; 3 Institute of Evolutionary Biology and Environmental Studies (IEU), University of Zürich, Zürich, Switzerland; University of Tasmania, Australia

## Abstract

**Background:**

Very few studies have ever focused on the elephants that are wounded or killed as local communities attempt to scare these animals away from their settlements and farms, or on the cases in which local people take revenge after elephants have killed or injured humans. On the other hand, local communities live in close proximity to elephants and hence can play a positive role in elephant conservation by informing the authorities of the presence of injured elephants.

**Methodology/Principal Findings:**

Between 2007 and 2011, 129 elephants were monitored in Masai Mara (Kenya), of which 54 had various types of active (intentionally caused) or passive (non-intentionally caused) injuries. Also studied were 75 random control samples of apparently unaffected animals. The observed active injuries were as expected biased by age, with adults suffering more harm; on the other hand, no such bias was observed in the case of passive injuries. Bias was also observed in elephant sex since more males than females were passively and actively injured. Cases of passive and active injuries in elephants were negatively related to the proximity to roads and farms; the distribution of injured elephants was not affected by the presence of either human settlements or water sources. Overall more elephants were actively injured during the dry season than the wet season as expected. Local communities play a positive role by informing KWS authorities of the presence of injured elephants and reported 43% of all cases of injured elephants.

**Conclusions:**

Our results suggest that the negative effect of local communities on elephants could be predicted by elephant proximity to farms and roads. In addition, local communities may be able to play a more positive role in elephant conservation given that they are key informants in the early detection of injured elephants.

## Introduction

Human-elephant conflict (HEC) is a chronic problem that occurs wherever elephants and people share habitat. This conflict is considered by the IUCN Species Survival Commission’s African Elephant Specialist Group (AfESG) as a major threat to the long-term survival of the African elephant. Human-elephant conflict can be defined generically as “any human-elephant interaction which results in negative effects on human social, economic or cultural life, on elephant conservation or on the environment” [1]. Even so, most studies are focused on the first premise of this definition, that is, the negative effects on human social, economic and cultural life [2] and little is known of the negative effect of these conflicts on elephant conservation [2]. HEC is a problem that poses serious challenges to wildlife managers, local communities and elephants alike [2] and occurs throughout the species’ range in Africa, both in forest ecosystems in west and central Africa [3] and savanna ecosystems in east and south Africa [4], [5]. Local communities in Kenya usually live in close proximity to elephants and are able to observe rapidly the presence of injured elephants and report such cases to the authorities; these people can hence play an important role as key informants in cases of elephant injury and participate positively in HEC. We report here the findings of the first study of the spatio-temporal distribution of injured elephants in Masai Mara and the putative negative and positive roles of the local community therein.

HEC has become an increasingly significant issue as human populations have expanded and encroached upon elephant habitat [6], [7]. Some of the major conflict areas in the Masai Mara ecosystem include the community group ranches around Masai Mara National Reserve such as Siana, Koiyaki, Lemek and Olderkessi-Naikarra and further north around Ntulele and Siyiapei [8].

Although the nature of the physical harm inflicted on free-ranging African elephants (*Loxodanta africana*) in Kenya is well documented [9], its spatio-temporal distribution and its relationship to human-elephant conflict has not been well studied. The main factor affecting the spatio-temporal prevalence of elephants is probably the seasonality of elephant movements and their relationship with local community settlements, farms, rivers and roads.

The Masai Mara ecosystem is home to the world famous Masai Mara National Reserve, which is characterized by intense human-wildlife-livestock interaction in the wildlife dispersal areas surrounding the Reserve [10]. The close interaction between people and wildlife have led to increased human-elephant conflicts within this animal’s dispersal areas and farmers and pastoralists alike respond by scaring elephants away from their farms and settlements using traditional weapons such as arrows and spears that can cause physical injuries to elephants. Some of these injuries are quite severe and if not treated can lead to the death or deformity of an elephant; hence in the long-term these lesions can have a real effect on elephant populations.

Free-ranging elephants are monitored by the Kenya Wildlife Service (KWS) for the presence of injuries (physical wounds or death). Wherever an injured elephant is detected, the KWS veterinarians immediately treat the injury or, if necessary, remove the carcass of the dead animal [9]. These interventions are time-consuming and entail high economic costs incurred by the KWS that include transport, drugs, darting equipment and personnel. Due to the severe and complex consequences of HEC, it is important to understand the factors that underpin the spatial occurrence of cases of harmed elephants in order to be able to formulate pragmatic mitigating responses.

The aims of this study were (i) to analyse the spatio-temporal distribution of injured elephants in Masai Mara between 2007 and 2011 and its possible relation to seasonality and proximity to local communities’ settlements, roads, rivers and farms, and (ii) to evaluate the possible positive role of local communities as key informants in the early detection of injured elephants.

## Methods

### Study Area Masai Mara Ecosystem

The Masai Mara ecosystem is located in southwest Kenya along the Kenya-Tanzania border (1°10’00″ and 2°10’00″ S, 34°14’50″ and 36°10’00″ E) [11]. The region is bounded by the Rift Valley to the east, the international border with Tanzania to the south, and the Siria Escarpment to the west. It includes the world-famous Masai Mara National Reserve (MMNR), a protected area for wildlife (about 1510 km^2^) along the border with Tanzania that is essentially the northern continuation of the Tanzanian Serengeti National Park. The MMNR is surrounded by community-owned group ranches (4870 km^2^), that act as wildlife dispersal areas in the north and east. Land uses on these ranches include traditional livestock pastoralism, wildlife conservation, tourism and a small amount of subsistence maize and wheat cultivation [11]. The Masai people living around MMNR depend on livestock for their livelihoods. Pastoral livestock farming (mainly goats, camels, cattle and sheep rearing) [12] is the dominant production system in this area, which is characterised by intensive wildlife-livestock-human interaction that includes the sharing of pasture and water. The Masai Mara ecosystem has dense populations of wildlife including large mammals such as African elephants, lions, leopards, African buffaloes, black rhinoceros, wildebeests and several antelope species. Rainfall in the Mara region is bimodal with a short rainfall period in November–December and a longer period in April–June. The long dry season spans July–October and the short dry season January–March. However, these seasons are not fixed and variations occur as the rains become less predictable [13].

Mean temperatures have risen in the Mara region in recent decades leading to progressive habitat desiccation [14]. In the period 1977–2009, this region also experienced severe recurrent droughts, the most noteworthy occurring in 1984, 1993, 1999–2000 [14], 2005–2006 and 2008–2009.

### Injured Elephants

**Figure 1:**
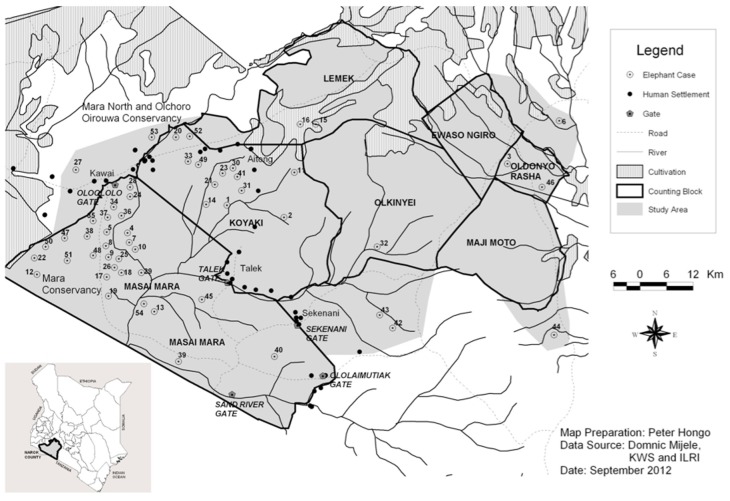
Spatial distribution of cases of elephant injury, rivers, roads, farmlands and human settlements in the Masai Mara ecosystem, Kenya.

Injured elephants (54 cases) were immobilized and georeferenced using hand-held GPS [15], [16] by KWS veterinarians for clinical treatment and biodata collection: age group, sex, georeference and the date of capture (Fig. 1). Elephants were classified by age as either sub-adult (<10 years) or adults (≥10 years). The nature of the injury, possible causes and the parts of the body affected were also recorded for each elephant. Injured elephants were immobilized by darting using a combination of etorphine hydrochloride (M99® Norvatis South Africa (Pty Ltd/(Edms) Bpk) and hyaluronidase at varying dosages depending on the age and sex of the injured elephant [15], [16]. The 1.5–3 ml darts, attached to a 60-mm long and 2.2 mm plain Dan-inject needles, were remotely delivered by a Dan-inject (Denmark) long-range projector. Immobilized individuals were then examined for injuries and the corresponding data recorded. Injuries were treated using 10% hydrogen peroxide, anti-inflammatory drugs and tincture of iodine and oxytetracycline spray (depending on the extent and location of the injury). In addition, long-term antibiotics were administered intramuscularly. After treatment the anaesthesia was reversed by the intravenous administration of diprenorphine hydrochloride [15].

**Figure 2:**
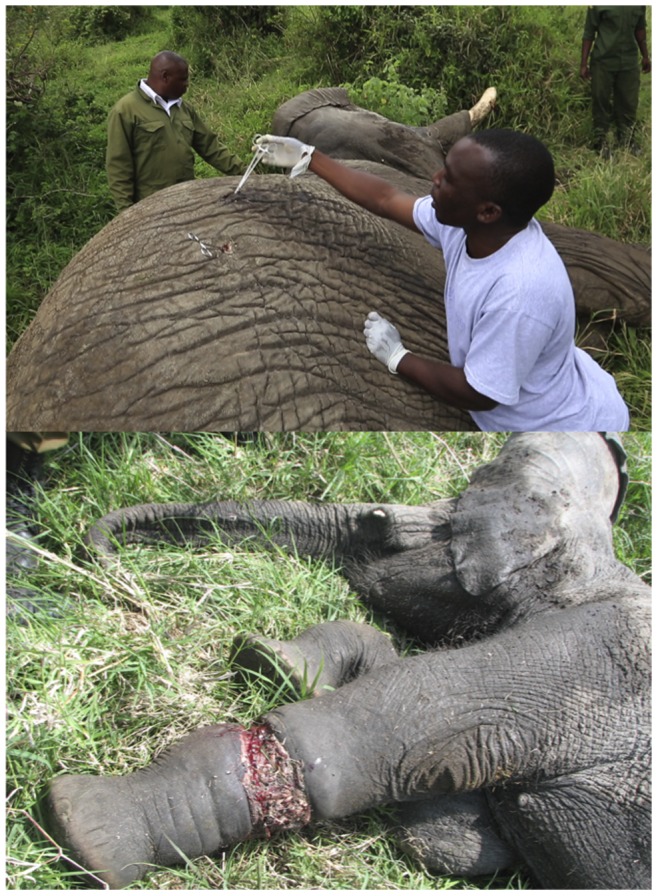
(Top) KWS vets treating an elephant actively injured with a poisoned arrow. (Below) A passively injured elephant in a snare. Masai Mara, Kenya. The two vets of the photograph have given written informed consent, as outlined in the PLOS consent form, to publication of their photograph.

The injured elephants were grouped into two categories: (i) actively injured elephants that had been intentionally attacked by the local communities using poisoned arrows or similar sharp objects (30 elephants), or (ii) passively injured elephants, which had been non-intentionally injured by the local communities via snares placed to capture wild animals for consumption as bushmeat (20 elephants). A further four injured elephants were not included in the analyses because we were unable to determine whether their injuries were active or passive (Fig. 2).

The response to cases of elephant injury was rapid since there is a resident KWS veterinarian in Masai Mara employed to deal with such occurrences; as well, the injuries generally greatly weakened the elephants and affected and/or reduced their ability to move. The pain caused elephants to remain close to where they had sustained their injuries. The distance moved after being wounded is normally short and we assume that it did not affect the distribution pattern of injury cases.

### Elephant Population Estimation

The distribution and population of healthy (non-injured) elephants in the Masai Mara ecosystem was estimated in 2010 from the total aerial counts described by Norton Griffins [17]. This involved the use of a fixed upper wing Cessna 182 four-seater aircraft. A Geographical Positioning System (GPS) was used for navigation and marking the locations of the elephants counted. The census was done at 1-km intervals in an east-west direction from a flying height of 100 m. Wherever large elephant herds were encountered, the aircraft circled the area to establish the exact herd size.

Besides the injured elephants, 75 non-injured, apparently healthy elephants were selected randomly from the study area for statistical analysis. All injured elephants were georeferenced using hand-held GPS and the coordinates were entered in an Arc-GIS to generate a spatial map. Human settlements, crop farms, rivers and roads in the study area were also mapped using an Arc-GIS. The distances between each elephant (either non-affected or passively or actively injured) and the nearest human settlement, crop farm, river and road were estimated (Fig. 1).

The calculation of the exact distribution of unaffected elephants in different periods (year and season) of our study was not possible. We assumed that the movement of elephants in Masai Mara is limited and that the snapshot sample that we carried out is representative of the rest of the study period.

### Data Analyses

To estimate the possible effect of human settlements, crop farms, rivers and roads as possible risk factors affecting elephant status (unaffected, or passively or actively injured) we used a GLM Multinomial Logistic Regression. In the first Multinomial Model all possible variables (distances from human settlements, crop farms, rivers and roads) and their interactions were included. The response variable was elephant status (unaffected, or passively or actively injured). This full model was simplified step-by-step by deleting the non-significant variables or interactions. The criteria for simplifying the model were based on AIC criteria and an ANOVA analysis between the two models. Given that the data corresponding to unaffected elephants did not distinguish between sex, age class or season as possible risk factors (given that the location of each elephant was based on aerial counts), we performed a Logistic Regression Analysis with as the response variable only actively and passively injured elephants, and as explicatory variables the distance from human settlements, crop farms, rivers and roads, elephant sex and age class (adults or sub-adults), and season (dry or wet). The first Logistic Regression Model included all the explicatory variables and their interactions and the simplifying procedure was the same as for the Multinomial Model process. Fisher’s Exact Test was applied to test the differences between season, sex and age class for actively and passively injured elephants. We did not consider crop-raiding reports from the communities around Mara in our analyses as a possible risk factor for elephant status since we had no appropriate systematic data; likewise, most cases of crop-raiding are not reported to the KWS and people merely chase elephants away, which sometimes get injured in the process, before they can raid crops. We used the R Package V.2.15.1 for all statistical analyses and figures.

### Ethics

The study was approved by the ethics committee of the Kenya Wildlife Service (KWS) and the Government Department of Veterinary Services of Kenya. KWS guidelines on Wildlife Veterinary Practice-2006 were followed. All KWS veterinarians follow the Veterinary Surgeons and Veterinary Para-Professionals Act 2011, Laws of Kenya, which regulates veterinary practices in Kenya.

## Results

In 2007–2011 in Masai Mara a total of 54 cases of injured elephants were detected and then examined and treated by a veterinarian. The injured elephants were classified into two categories: 60% (30/50) were actively injured elephants that had been intentionally attacked by local people with poisoned arrows or similar sharp objects, while 40% (20/50) were passively injured that had been non-intentionally injured by local people in snares placed to capture wild animals as bushmeat. Four other injured elephants were not included in the analyses because we were unable to ascertain the origin (active or passive) of their injuries (Fig. 2). There were no repeat injuries in our study.

**Figure 3:**
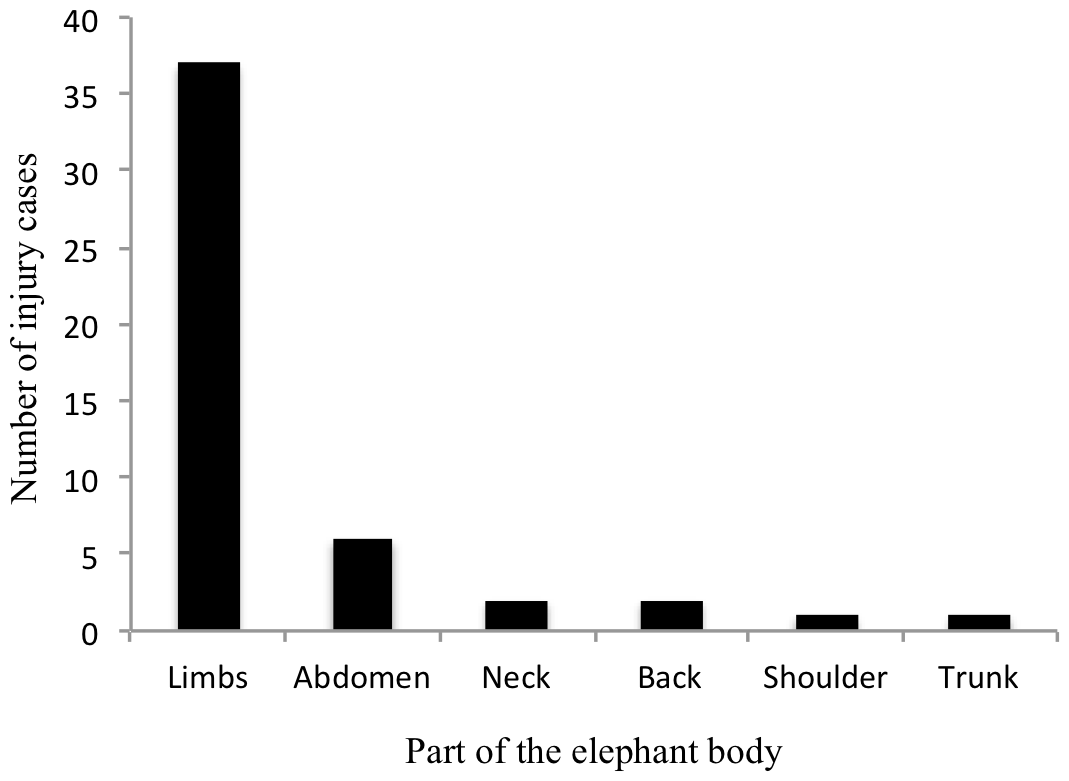
Number of injury cases involving different elephant body parts in 2007–2011 in the Maasa Mara, Kenya.

**Figure 4:**
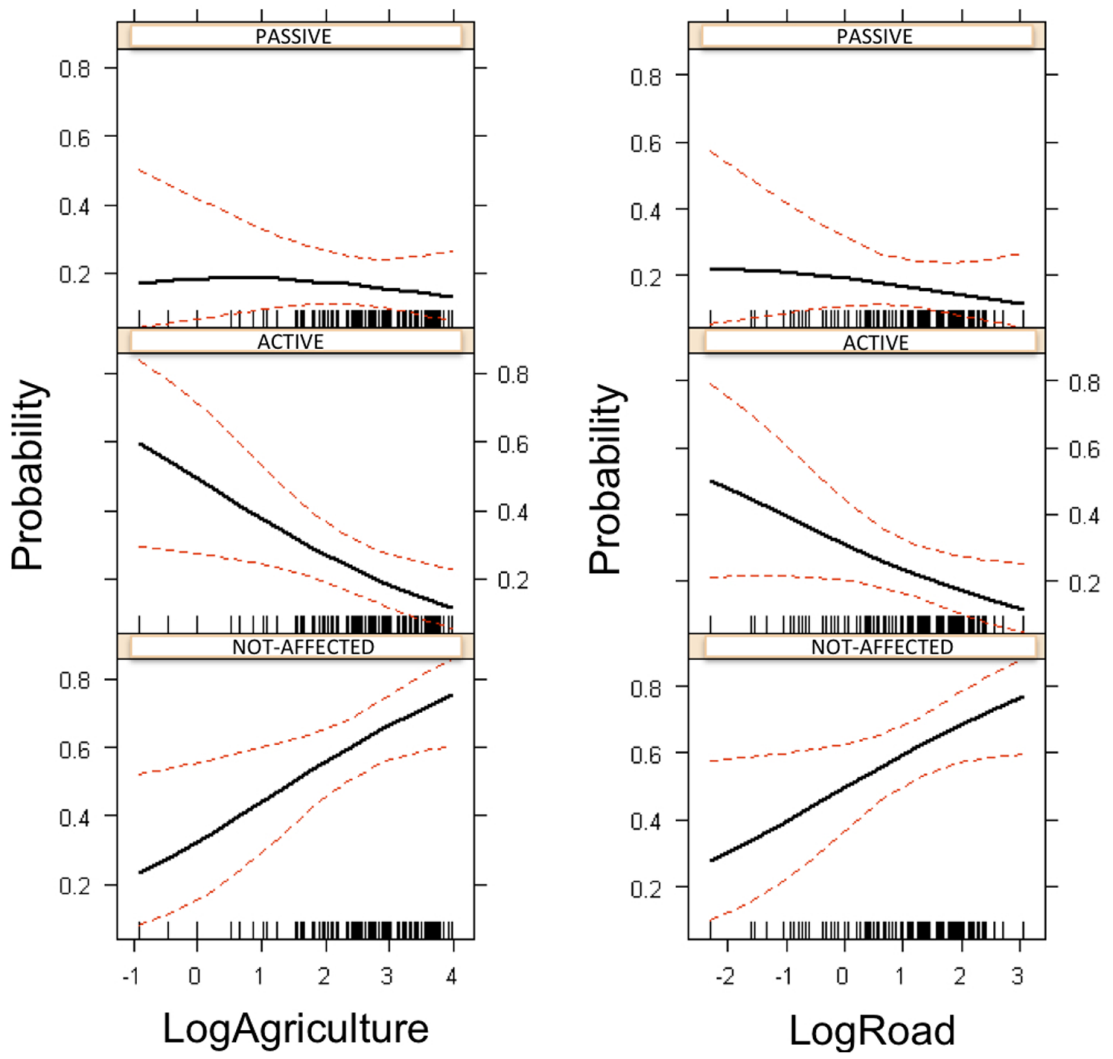
The effect of agricultural lands and roads on active and passive elephants injuries and unaffected elephants. This figure only includes 75 unaffected elephants, when the real number is 3,072 elephants.

Elephant limbs were the most vulnerable body part to injuries (Fig. 3). A Multinomial GLM indicated that road (*p*<0.015) and agriculture areas (*p*<0.001) had a negative effect on the health status of the elephants; on the other hand, neither human settlement nor water had any effect. The effect of agriculture areas and road had the same effect on passive and active injuries: the nearer the elephant to agriculture areas and road, the greater the possibility of being actively or passively attacked. The number of elephants with passive or active injuries increased in the proximity of agricultural areas and roads (Fig. 4).

The number of injured and healthy elephants was not significantly affected by the presence of water sources or human settlements. The selected Multinomial GLM was Injury (Active) ∼ 0.9434 (±0.6123) –0.5729(±0.2077) LogAgriculture −0.4617(±0.2098) LogRoad. Injury (Passive) ∼ −0.2197 (±0.7446) –0.2957(±0.2460) LogAgriculture −0.3113 (±0.2367) LogRoad.

When we considered only the injured (active and passive) groups, a GLM using the binomial model included only elephant age class as a significant variable (*p* = 0.020). Injury ∼ 0.9651(±0.4155) −1.2528(±0.6059) Age.

Adult elephants were more vulnerable to actively inflicted injuries: 70% (21/30) were adults while only 30% (9/30) were sub-adults (*p* = 0.038). This was not the case of passively affected elephants of which adults accounted for 40% (8/20) and sub-adults 60% (12/20).

Although not statistically supported (*p* = 0.55), males were proportionally more affected than females by passive injuries –14 males (70%) and 6 females (30%) – and by active injuries –18 males (60%) and 12 females (40%).

The highest number of injury cases in elephants (n = 15) occurred in 2008 and 2011, while the least number of cases (n = 2) were detected in 2007. The post-mortem examination of freshly dead carcasses (n = 5) revealed gross pathologies associated with inflicted injuries.

**Figure 5:**
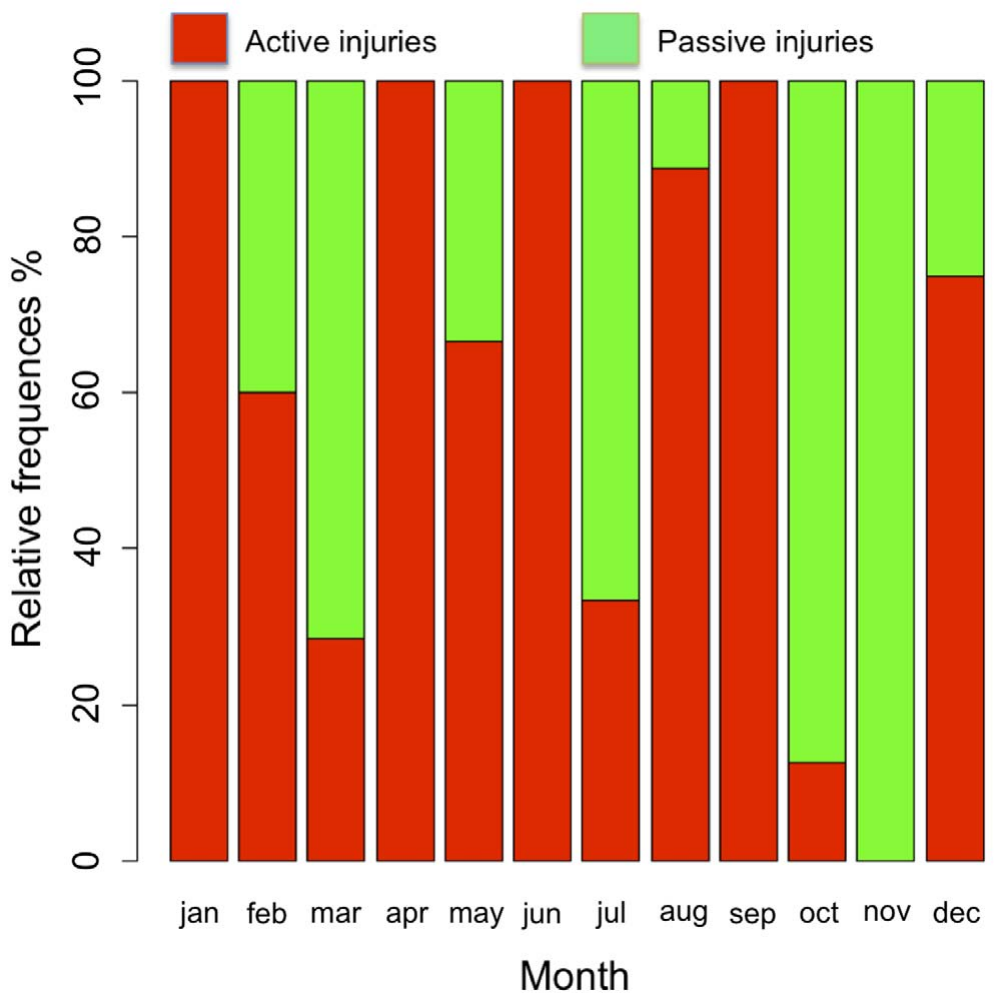
Monthly distribution of actively and passively injured elephant cases in Masai Mara, Kenya, between 2007 and 2011.

There was seasonal variation in the number of actively injured elephants; more cases of actively injured elephants were detected in the dry season (19; 63%) than in the wet season (11; 37%), even though the difference was not statistically supported (*p* = 0.43). There were no differences between dry and wet (both 10; 50%) seasons (Fig. 5) in the number of passively affected elephants.

During the study period local communities reported 43% (23/54) of the injured elephants, while KWS/County council rangers reported the other 57% (31/54) cases. Out of the 23 cases reported by the local community, four were passive cases (20%; 4/20 out of the total number of passive cases) and 19 were active cases (63%; 19/30, out of the total number of active cases).

## Discussion

The human elephant conflict (HEC) is often defined and assessed principally on the basis of the harm inflicted on people and/or their properties. However, local communities are known to inflict retaliatory injuries on elephants, some of which cause severe wounds and even death [9]. These conflicts are numerous in areas in which people and elephants share habitat because elephants forage widely beyond the boundaries of protected areas and enter into human settlements and crop farms [2], [18].

Cases of injured (physically injured or killed) elephants in the Masai Mara ecosystem are monitored by KWS veterinarians to decide whether or not intervention and/or treatment is necessary. Except for a few elephants that could not be traced in the wild due to the rugged terrain and the elephant’s large ranges, most cases were treated. Almost all elephants recovered after treatment and only 2/54 (3.7%) succumbed to injuries in the period after treatment. About five cases were reported for post-mortem examination.

HEC has been reported to occur in all areas where elephants’ ranges overlap with human settlements regardless of whether or not agriculture is practiced [2], [18].

Elephants are known to forage widely beyond the boundaries of protected areas and enter into cultivated crop farms. This crop-raiding behaviour is a risk factor [19] that frequently causes conflicts and results in elephant injury (physical injury or death) [9].

During the study period there were more actively caused than passively caused injuries. Passively caused injuries are not intentional and occur where local communities use snares to capture wild animals that do not target elephants specifically.

In our study males were more affected than females in cases of both active and passive injuries. Crop-raiding seems to be sex-biased towards males [20] and likely hinges on nutritional advantages that can enhance their fitness and reproductive competitiveness [21], [22]. This is because sexual selection in a polygynous species such as the elephant is biased towards dominance [23]. The propensity of male elephants to raid crops makes them vulnerable to human retaliatory attacks that can cause the high prevalence of injuries observed in males [9] and in our results. Crop-raiding is rare in females – even if they inhabit areas close to crop farms [21], [24] – and male elephants in Africa and Asia account for 70–100% cases of crop damage [25]–[27].

Our results suggest that adult elephants are more likely to be actively injured than young animals. This is probably due to the species’ complex social relationships characterized by tightly led matriarchal core units offering security to young elephants that contrast with flexible male units [28]. Young male and female elephants remain in the matriarchal herd and at the hint of danger adult females (even from other social units) rush to form a tight ring around the young animals [29]. Furthermore, matriarchal groups avoid risky behaviour such as crop raiding [21] and so young elephants sustain fewer injuries. This is not the case of passive injury, which affects adults and young elephants similarly; this is logical given that passive injury is only a question of bad luck and is not specific to any age class. Wire snares, usually set at ground level along animal tracks, are indiscriminate and target multiple species of wildlife [30], and may serve both as a retaliatory weapon and for illegal capture. Farmers tend to use objects and weapons such as arrows, spears or poisoned nails that injure elephants in more subtle ways than guns, which draw too much attention [9].

Most studies report that crop destruction is the most important economic damage inflicted by elephants on humans [2], [31], [32]. Farming communities near national parks and forested areas in Kenya report serious crop damage caused by elephants [33]. As a result, elephants are likely to be attacked by local communities and scared away from their farming areas before they can feed on their food crops (e.g. maize, bananas, cabbages, pumpkins and carrots) or destroy mature crops and inflict serious economic losses [2], [31], [33], [34]. Irigia [34] and Kabunge *et al.* [35] recorded crop losses of up to Kshs. 100,000 at Ol Ari Nyiro ranch in southern Ghana, while Barnes *et al.* [36] reported an average loss of 50% in some crops. Both passive and active injury cases had a significant negative correlation with crop farms (agriculture areas) indicating that elephants are more likely to be injured near crop farms than further away. The association of elephant injury and crop farms is indicative of HEC. The occurrence of injured elephants close to crop farms is suggestive of habitual crop-raiding by elephants. Our results concur with previous studies that suggest that injury cases were male dominated [9], which reflects the male bias in crop-raiding. The fact that many elephants in the Mara ecosystem concentrate near crop farms could be because crop-raiding in savannah ecosystems is triggered when the quality of wild grasses declines below the quality of crop species [37]; in forest ecosystems the availability of mature crops influences the extent of crop raiding [19]. This also explains why most of the active injuries were recorded during the dry months of the year.

Seasonal changes in the distribution of food resources have an impact on the spatial structure, demography and movement patterns of mega-herbivores such as elephants [38], [39]. A seasonal variation occurred in the number of active injury cases: there was a relatively high number of cases in February and March in the dry period, just before the rainy crop-planting season begins in April–June, which is when elephants are ranging furthest in search of food and water. They are likely to pass through people’s homesteads and property and so there are greater chances of conflict with villagers. The higher number of cases in August–October could be attributable to harvesting and the low rainfall season during which elephants raid farms and share watering points with livestock and people. The chances of being attacked by people and sustaining traumatic injuries are high during this period in cultivated areas within the Masai Mara ecosystem. No seasonal pattern was observed in the case of passively caused injuries indicating that local communities use wire snares with the same intensity in the dry and wet seasons.

Water is an important resource in the life of elephants and influences their spatial distribution in the landscape since they require water for drinking and mud-bathing on a daily basis [33], [40]. In the present study, water sources (rivers) did not significantly affect the spatial distribution of injured elephants in the Masai Mara ecosystem but it was observed that, irrespective of injury, elephants tend to concentrate near water bodies. In habitats that are intensely poached, surface water bodies are risk areas for elephants, above all in the dry seasons [41], [42].

Human population and settlements have increased in the Masai Mara ecosystem and have expanded into wildlife conservation rangelands [43]. As our results indicate, elephants concentrate close to human settlements, probably due to the inadequate buffers between elephants and these settlements.

The negative effect of roads on cases of elephant injuries can be explained by the fact that many incidences of HEC and elephant attacks occur along roads when people accidentally encounter elephants. Elephants had a greater possibility of being injured when they were near roads and so most injury cases were reported close to roads. Another factor to take into account are the extensive road networks within the reserve and the immediate surroundings due to increased human activity and the construction of roads for tourist vehicles. Generally, more elephants (injured or non-injured) were found close to roads because roads are built near elephant ranges (rather than there being any tendency for elephants to approach roads). Many roads have permeated into elephant rangelands and elephant ranges are now more accessible than before.

Our study was limited to the physical consequences of human attacks on elephants and more studies are still needed to evaluate the effect of these attacks on responses in elephant such as (i) attack/injury, (ii) behaviour (movement dynamics) and (iii) physiology (stress hormone metabolite concentrations) [44].

Local communities do not have merely negative attitudes regarding the presence of elephants and can play a pivotal role in conservation due to their direct contact with elephants and their ability to inform authorities if they observe injured animals. Curiously, local communities reported a greater proportion of active cases (63% of active cases) than passive cases (20% of passive cases). Obviously, the community members who report cases are not the culprits and so report all cases to the KWS vets without fear. Active cases, usually caused by arrows, could be more visible than passive cases, which are usually the result of placing snares. This again, highlights the importance of well-informed communities in the conservation of wild animals [12].

### Conclusions

The different types of human-elephant conflicts, in which elephants are attacked, injured or even killed by local communities, are still neglected. Likewise, the positive role of local communities as key informants in the early detection of the injured elephants is still not fully appreciated. Our results suggest that local communities inflict active injuries on elephants in retaliation for the destruction of their properties or deaths. However, the concentration of actively injured elephants closer to crop farms and roads and away from settlements suggests that injured elephants are likely to risk repeat raids near the road network. Injured elephants are unlikely to risk remaining close to human settlements. This suggests that the presence of crop farms and roads in elephant areas is a high risk factor driving the incidence of HEC, the prevalence of injury cases and the spatial distribution of injured elephants. Local communities may also negatively affect elephants by their use of snares to capture other wild animals as bushmeat. Nevertheless, local communities do play positive roles as key informants in the early detection of injured elephants. More efforts should be made to safeguard elephants in parts of the Masai Mara ecosystem, especially in close proximity to crop farms and roads, and attempts should also be made to raise awareness in local communities and encourage them to play their parts in saving the elephants. This would reduce elephant injuries and mortalities related to the human-elephant conflict and save the cost of chemical immobilization and treatment of affected elephants.
